# Bibliometric analysis of nutrition in gastric cancer from 2013 to 2023

**DOI:** 10.3389/fnut.2024.1402307

**Published:** 2024-09-18

**Authors:** Ruyin Li, Zirui Zhao, Hongyun Huang, Jianchun Yu

**Affiliations:** ^1^Department of General Surgery, Peking Union Medical College Hospital, Peking Union Medical College and Chinese Academy of Medical Sciences, Beijing, China; ^2^Department of Neurology, Peking Union Medical College Hospital, Beijing, China

**Keywords:** bibliometric analysis, nutrition, gastric cancer, nutrition therapy, nutritional status

## Abstract

**Background:**

Increasing evidence suggests that nutrition plays an important role in the treatment of gastric cancer. However, no bibliometrics analysis has been conducted in this field. Our study aimed to conduct a bibliometric study to explore the latest publishing trends and areas of intense activity within the sphere of nutrition in gastric cancer.

**Method:**

Publications were extracted from the Web of Science Core Collection. CiteSpace (Version 6.2.4) and VOSviewer (Version 1.6.18) were used for visual analysis.

**Results:**

In total, there were 441 publications authored by 2,941 authors from 809 organizations and 47 countries, published in 182 journals from 2013 to 2023. The most prolific country was China, and the most productive institution was the Chinese Academy of Medical Sciences. The leading core journal was Nutrients. P Daisuke Kobayashi and Yasuhiro Kodera were the most influential authors. The first highly cited document was published in Gastric Cancer by Kamarajah et al. The hotspots in this field were nutrition treatment and nutritional status. Moreover, research on nutritional status and nutrition-related prognosis in gastric cancer might be a potential trend.

**Conclusion:**

Nutrition in gastric cancer is a burgeoning research field garnering increasing attention. Further investigation is necessary to better understand the impact of nutritional status on the prognosis of gastric cancer.

## Introduction

Gastric cancer (GC) is a digestive cancer with a poor prognosis, imposing a huge burden on both society and the healthcare system (1–3). In recent years, researchers have conducted numerous studies on the prognosis and nutrition related to GC (4–7). These studies have shown that evaluating patients’ nutritional status and implementing nutritional support therapy have a beneficial impact on the treatment of GC (8–12). These encouraging results have attracted increasing attention to this field (7, 13). However, to date, no bibliometric analysis of recent literature on nutrition in GC has been published. This study aims to fill this gap by examining scientific publications in this field over the past decade.

To gain a deeper understanding of the forefront and focal points of nutrition research in GC, we need to address the following questions:

1. What has been the trend in annual publication over the past decade?
2. Which countries, institutions, and journals are the most productive and influential? How do they interconnect?
3. Who are the most prolific authors, and what are the focuses of their research?
4. What are the most frequently searched keywords and burst words? What are the primary research directions for emerging trends? How have these predominant research directions evolved over time?
5. What are the most cited documents, and what are their main contributions?

## Materials and methods

### Data and search strategy

English articles classified as ‘article’ and ‘review’ related to GC Nutrition from 2013 to 2023 were retrieved from the Web of Science Core Collection. It was important to note that the search terms “gastric cancer” and “nutrition” correspond to their respective entries in the Medical Subject Headings (MeSH) thesaurus used in the search query.

The total search strategy was as follows:(((TS = (Nutritional Processes OR Nutrition, Enteral OR Status, Nutrition OR Nutrition, Parenteral OR Undernutrition OR Support, Nutritional OR Nutrition, Total Parenteral OR Nutritional Intakes OR Nutrition Assessments, Mini OR National Health and Nutrition Examination Survey OR Protein Calorie Malnutrition OR Nutritional Disorder OR Quality, Nutritional Food OR Nutritional Physiology, Child OR Parenteral Nutrition Solutions OR Therapy, Medical Nutrition OR Nutrition Requirements OR Healthy Nutrition OR Nutritional Science OR Genetics, Nutritional OR Genetics, Nutritional OR Sciences, Exercise Nutritional OR Plant Based Nutrition OR Nutrition, Home Parenteral OR Elder Nutrition Physiological Phenomena OR Diet, Food, and Nutrition OR Nutrition, Home Total Parenteral)) AND TS = (Gastric Cancer)) AND DT = (Article OR Review)) AND LA = (English) (From January 1, 2013 to December 31, 2023) (retrieved March 1, 2024).

### Method

After experienced clinicians reviewed the abstracts of retrieved articles to exclude irrelevant ones, bibliographic information for the selected literature was downloaded. CiteSpace (Version 6.2.4) and VOSviewer (Version 1.6.18) were applied to analyze these documents visually (14, 15). VOSviewer was used for analyzing countries, institutions, authors, and citations, while CiteSpace was utilized for deduplicating and analyzing keywords and burst words.

**Figure 1:**
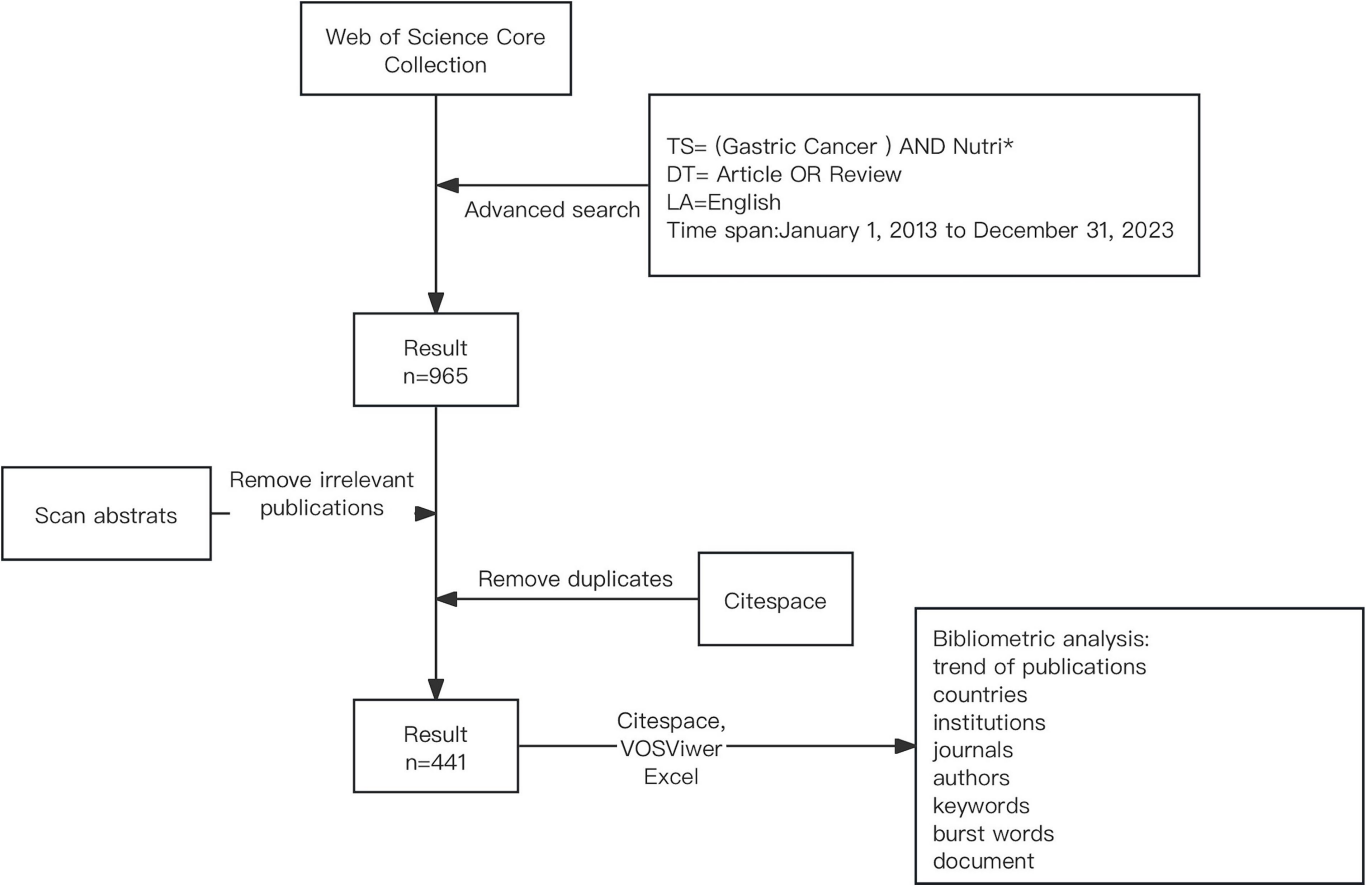
Detailed flowchart steps of the search strategy and bibliometric analysis.

Detailed flowchart steps of the search strategy and bibliometric analysis are shown in Figure 1.

## Results

A total of 441 relevant documents were identified, comprising 375 articles and 66 reviews authored by 2,941 individuals from 809 institutions, published across 182 journals and originating from 47 countries.

### Trend of publications

**Figure 2:**
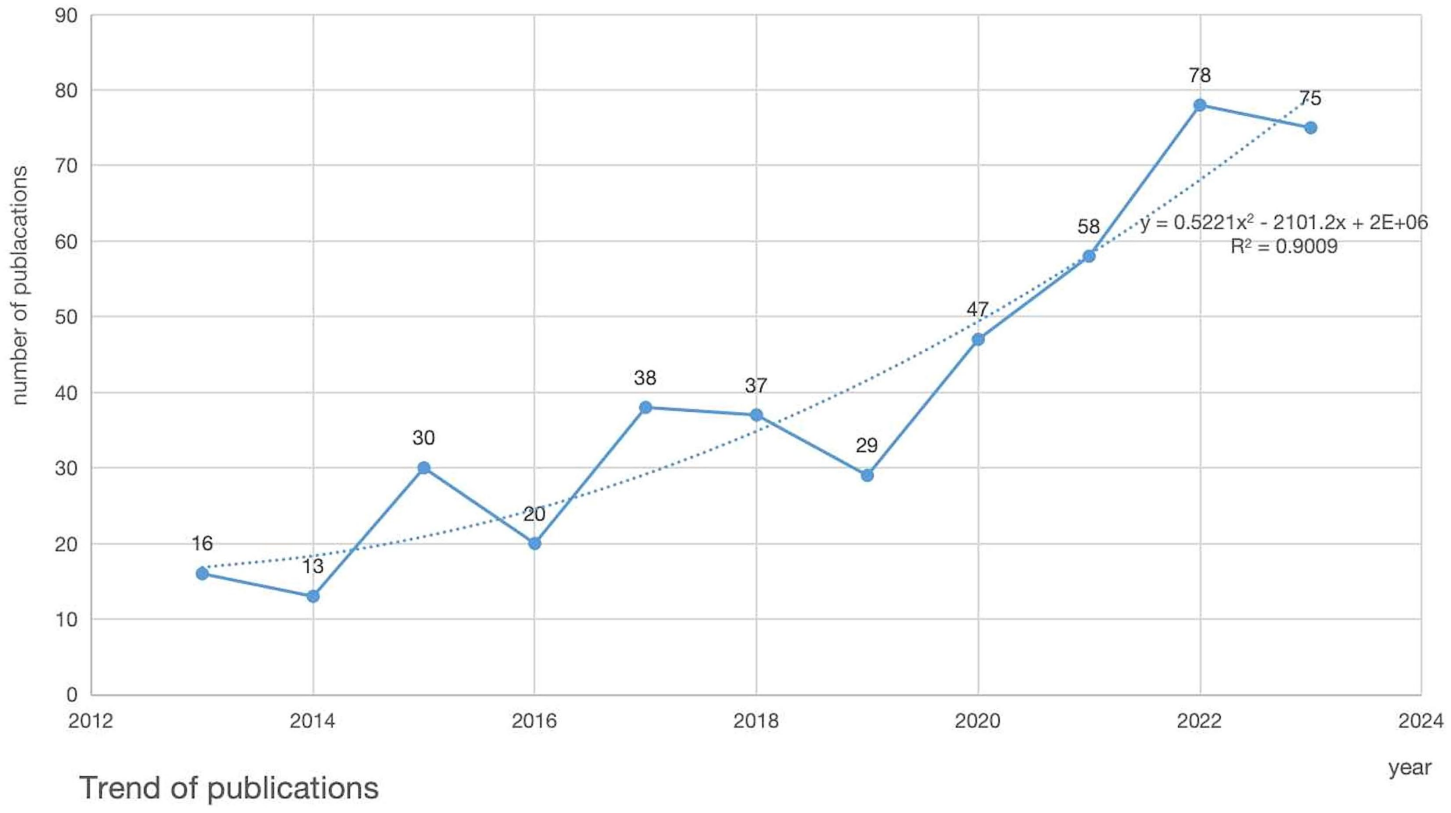
Trend of publications.

A total of 441 articles have been published in the field of GC nutrition. Figure 2 depicts the trend of annual publications. It can be observed from Figure 2 that the number of publications increased significantly from 2019. Based on the polynomial prediction curve for the annual publications ($y=0.5221x^{2}-2101.2x+2E+06,R^{2}=0.9009$), the volume of publications is expected to continue rising consistently in the coming years.

### Countries

**Figure 3:**
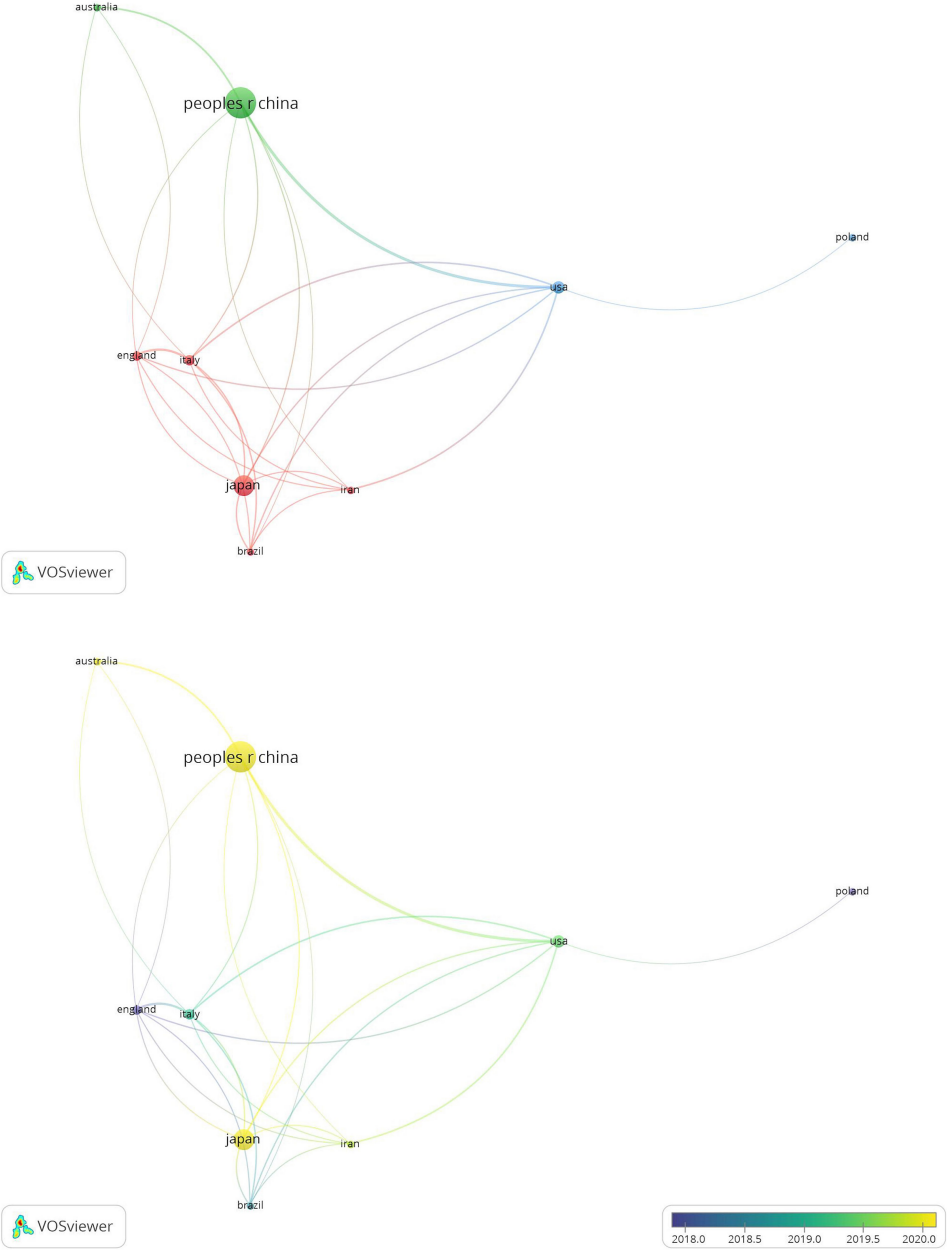
Network and overlay mapping of the top 10 countries.

The 441 articles included in this study originated from 47 countries. By setting the minimum occurrence to 11, the top 10 countries with the most publications were obtained (Table 1). It suggested that China was the most productive country, contributing 189 publications, which accounted for more than one-third of the total publications. Japan followed with 85 articles and South Korea with 44 articles. England had the highest citations per article at 32.47, followed by Italy with 21.55 and the USA with 19.3. Figure 3 displays the visualization of the cooperation network and overlay mapping among the aforementioned countries. It was clear that, except for South Korea, there was collaboration among the remaining nine countries. Furthermore, these countries could be categorized into three groups or clusters. The overlay mapping indicated that since 2020, China, Japan, and Australia have been the primary publishing countries in this field.

**Table 1:** Top 10 countries.

Rank	Country	Publication	Citation	Citation/publication
1	China	189	2,698	14.28
2	Japan	85	1,558	18.33
3	South Korea	44	614	13.95
4	USA	30	579	19.3
5	Italy	22	474	21.55
6	England	19	617	32.47
7	Australia	13	192	14.77
8	Iran	13	191	14.69
9	Brazil	12	206	17.17
10	Poland	11	110	10

### Institutions

**Figure 4:**
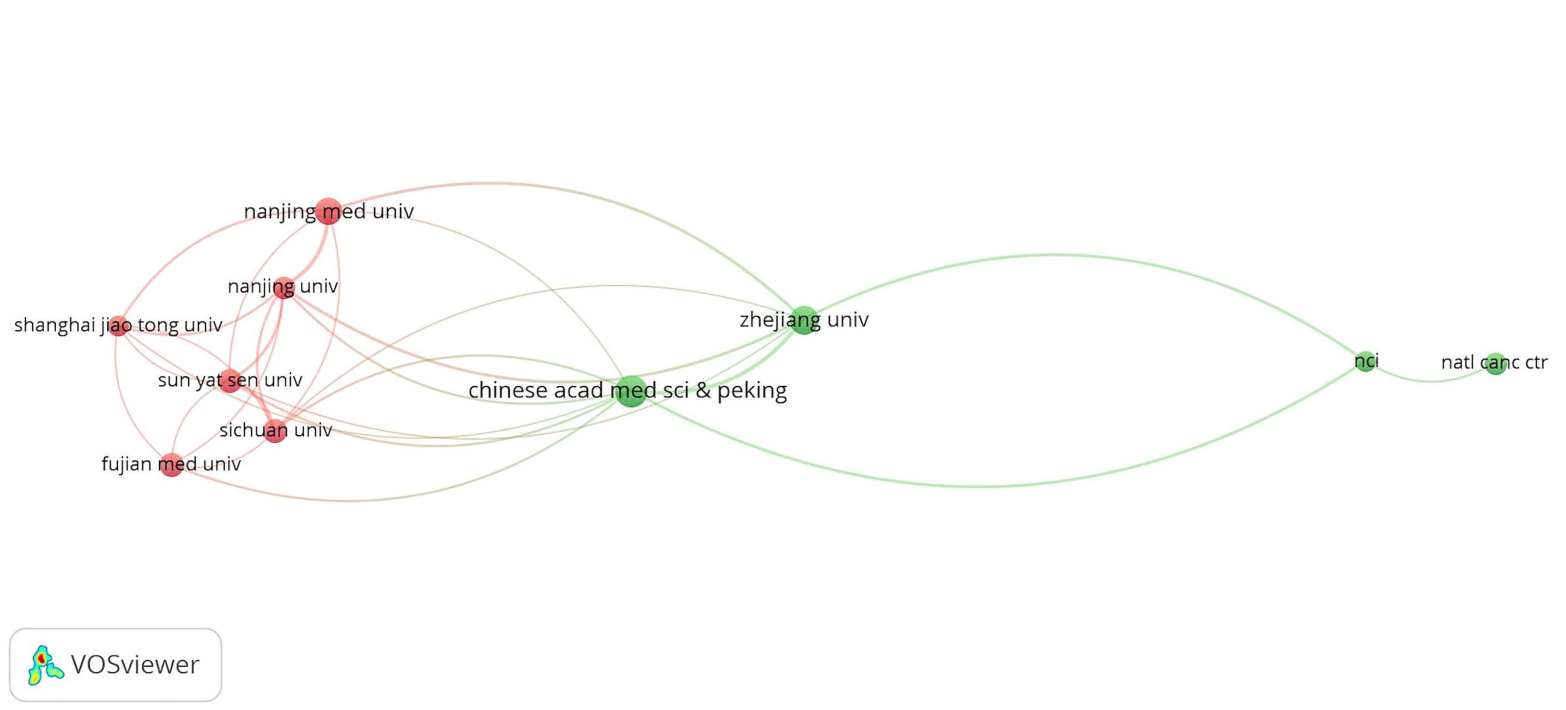
Network mapping of the top 10 organizations.

A total of 809 institutions were involved in this study. Table 2 displays the top 10 productive institutions in GC nutrition, with a minimum publication threshold set to 8. It revealed that the top three productive organizations were the Chinese Academy of Medical Sciences, Zhejiang University, and Nanjing Medical University, with 19, 17, and 15 publications, respectively. Nanjing Medical University had the highest citations per article, with 24. Among the 10 most scientifically productive institutions, 8 were from China and 2 from the United States. Figure 4 illustrates the collaborations and publication timelines, showing that collaborations can be categorized into two clusters. Furthermore, the Chinese Academy of Medical Sciences has emerged as the leading institution since 2021.

**Table 2:** Top 10 institutions.

Rank	Institutions	Publications	Citation	Citation/publication	Country
1	Chinese Acad Med Sci & Peking Union Med Coll	19	144	7.58	China
2	Zhejiang Univ	17	336	19.76	China
3	Nanjing Med Univ	15	151	10.07	China
4	Sichuan Univ	12	203	16.92	China
5	Sun Yat Sen Univ	11	261	23.73	China
6	Fujian Med Univ	11	116	10.55	China
7	Nanjing Univ	10	240	24	China
8	Natl Canc Ctr	10	130	13	USA
9	NCI	8	114	14.25	USA
10	Shanghai Jiao Tong Univ	8	112	14	China

### Authors

A total of 441 articles with 2,941 authors were incorporated in the study. By setting the occurrence threshold to 5, the top 10 productive authors in GC nutrition could be identified (Table 3). All of them were influential authors in this field. As shown in Table 3, from 2013 to 2023, the maximum number of publications by authors was 6, and the most productive authors were Daisuke Kobayashi, Yasuhiro Kodera, Carlo La Vecchia, Souya Nunobe, and Weiming Kang, with 32.17, 32.17, 21, 9.67, and 3.5 citations per article, respectively. The other authors each had published five publications, which Michitaka Fujiwara, Mitsuro Kanda, Kenta Murotani, and Chie Tanaka had each published five articles, with an average citation count of 30.6 per article.

**Table 3:** Top 10 authors.

Rank	Name	Publication	Citation	Citation/publication
1	Kobayashi, Daisuke	6	193	32.17
2	Kodera, Yasuhiro	6	193	32.17
3	La vecchia, Carlo	6	126	21
4	Nunobe, Souya	6	58	9.67
5	Kang, Weiming	6	21	3.5
6	Fujiwara, Michitaka	5	153	30.6
7	Kanda, Mitsuro	5	153	30.6
8	Murotani, Kenta	5	153	30.6
9	Tanaka, Chie	5	153	30.6
10	Yang, Jie	5	123	24.6

### Journals

The 441 articles in this study were sourced from 182 journals. According to Bradford’s law, the ratio of the number of core, related, and peripheral journals in a field at a given time conforms to 1:n:n2 (16). Thus, there were approximately 13 core journals in this field. By setting the minimum number of publications to 6, the core journals were identified (Table 4). According to Table 4, the top three journals were Nutrients, Nutrition and Cancer—An International Journal, and Annals of Surgical Oncology, publishing 25, 23, and 11 articles, respectively, with average citations of 6.72, 12.78, and 57.36 per article. In addition, Annals of Surgical Oncology, Gastric Cancer, and the European Journal of Surgical Oncology (EJSO) were influential journals noted for their high average number of citations.

**Table 4:** Core journals.

Rank	Source	Publication	Citation	Citation/publication
1	Nutrients	25	168	6.72
2	Nutrition and Cancer-an International Journal	23	294	12.78
3	Annals of Surgical Oncology	11	631	57.36
4	Clinical Nutrition	10	331	33.1
5	Supportive Care in Cancer	10	223	22.3
6	Gastric Cancer	9	489	54.34
7	Medicine	9	249	27.67
8	Frontiers in Nutrition	9	40	4.44
9	EJSO	6	316	52.67
10	PLOS One	6	114	19
11	BMJ Open	6	30	5
12	Frontiers in Oncology	6	29	4.83
13	Frontiers in Surgery	6	8	1.33

### Keywords

**Figure 5:**
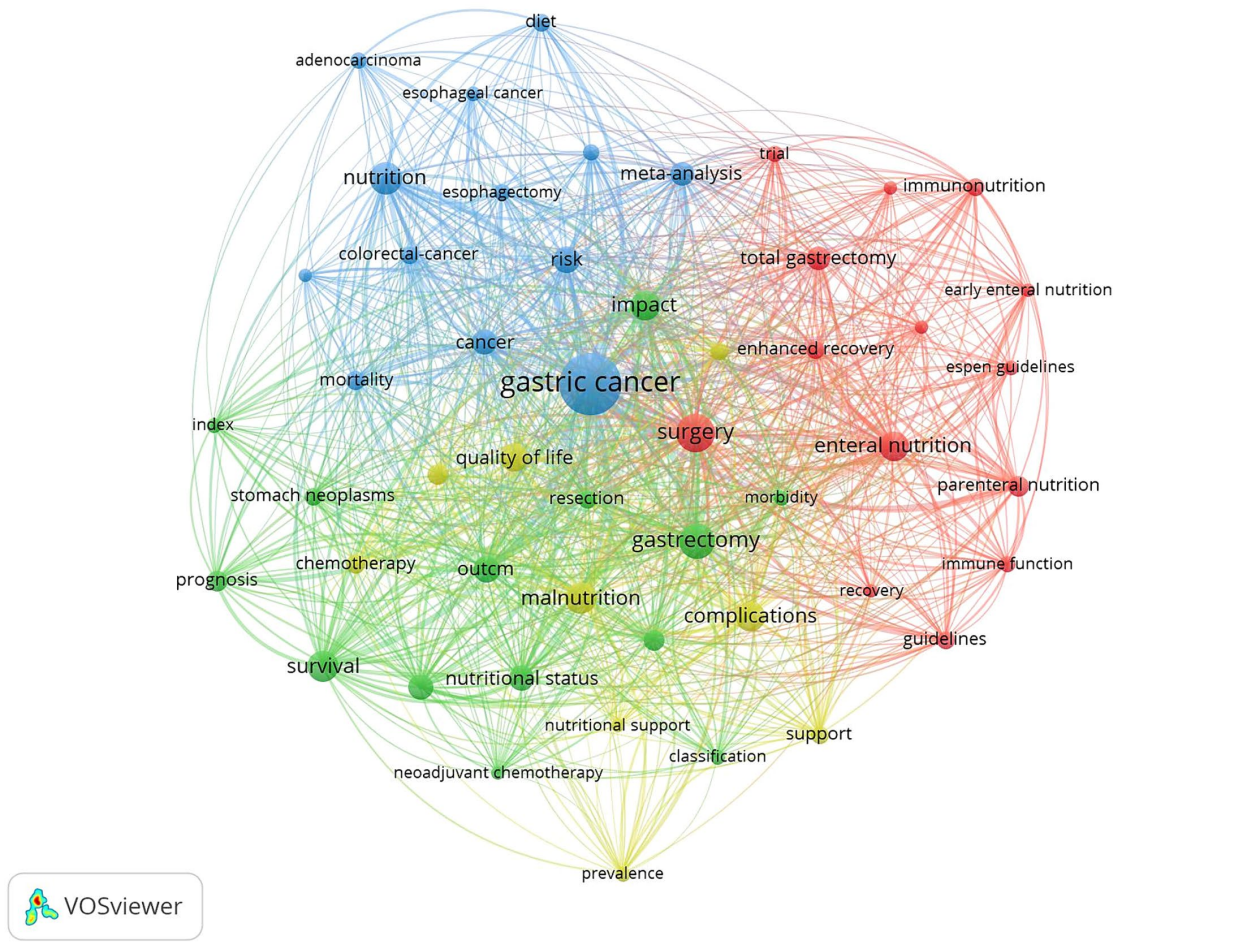
Overlay mapping of the most frequent 50 keywords.

**Figure 6:**
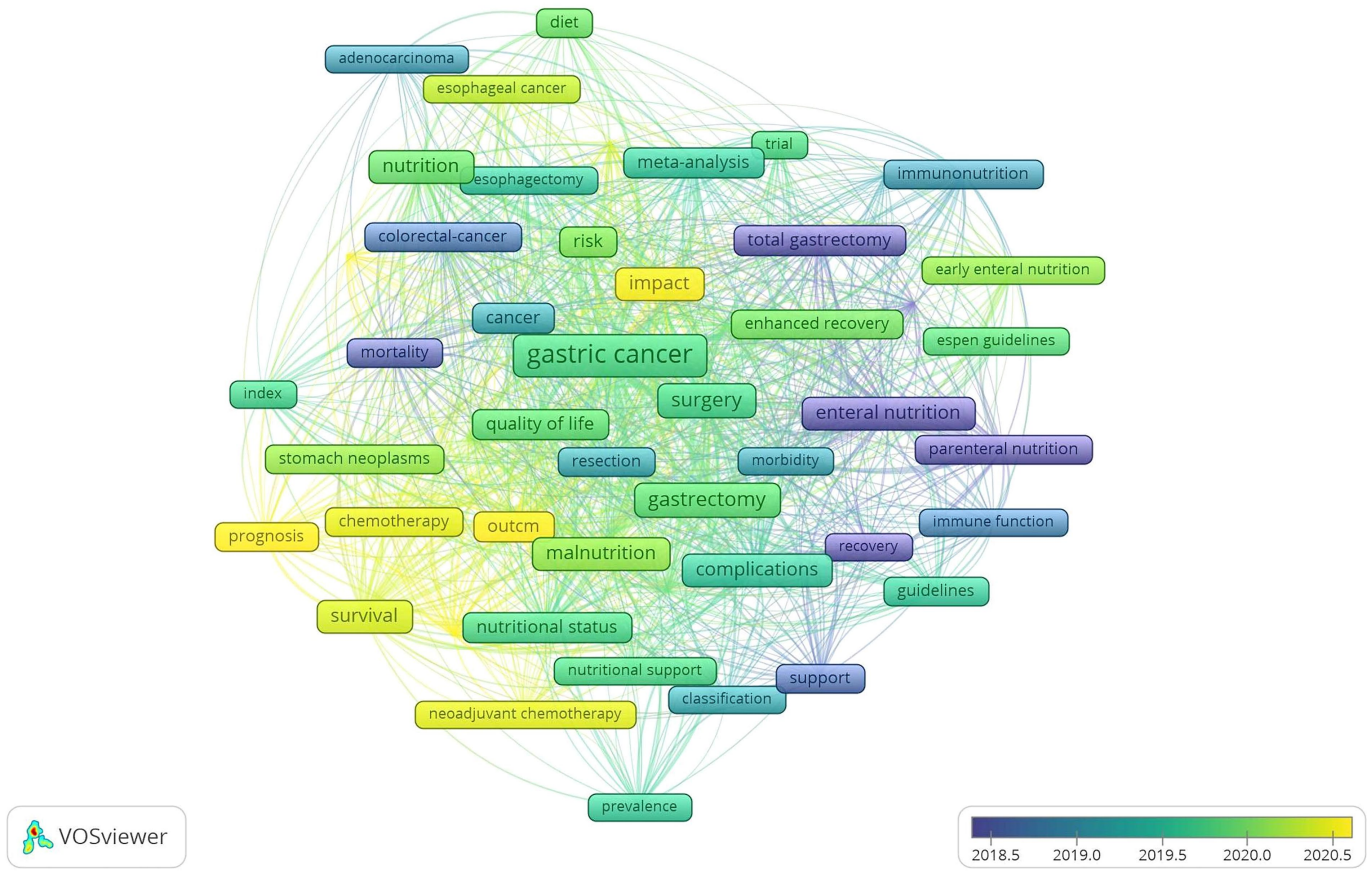
Overlay mapping of the most frequent 50 keywords.

A total of 1,690 keywords were extracted from 441 articles. By setting the occurrence threshold to 14 the relationships among the top 50 ranked keywords were analyzed (Figure 5). These keywords were categorized into four clusters: The red cluster related to surgery and postoperative nutrition support the blue cluster related to other tumors and risk factors and the green and yellow cluster focused on nutritional status postoperative complications and prognosis. In addition Figure 6 overlays these 50 keywords showing a shifting research trend in GC nutrition from surgery and postoperative enteral and parental nutrition support to complications nutritional status and prognosis. By setting the minimal occurrence to 32 the most important 20 keywords could be derived (Table 5). These keywords encompassed various aspects including surgery nutrition support nutritional status outcome and prognosis

**Table 5:** The 20 most frequently used keywords.

Keyword	Occurrence	Keyword	Occurrence
Gastric cancer	265	Outcm	51
Surgery	103	Risk	51
Gastrectomy	91	Cancer	47
Nutrition	75	Nutritional status	44
Malnutrition	68	Sarcopenia	44
Survival	67	Meta-analysis	43
Impact	64	Total gastrectomy	41
Enteral nutrition	63	Parenteral nutrition	33
Complications	60	Postoperative complications	32
Quality of life	55	Weight-loss	32

### Burst words

**Figure 7:**
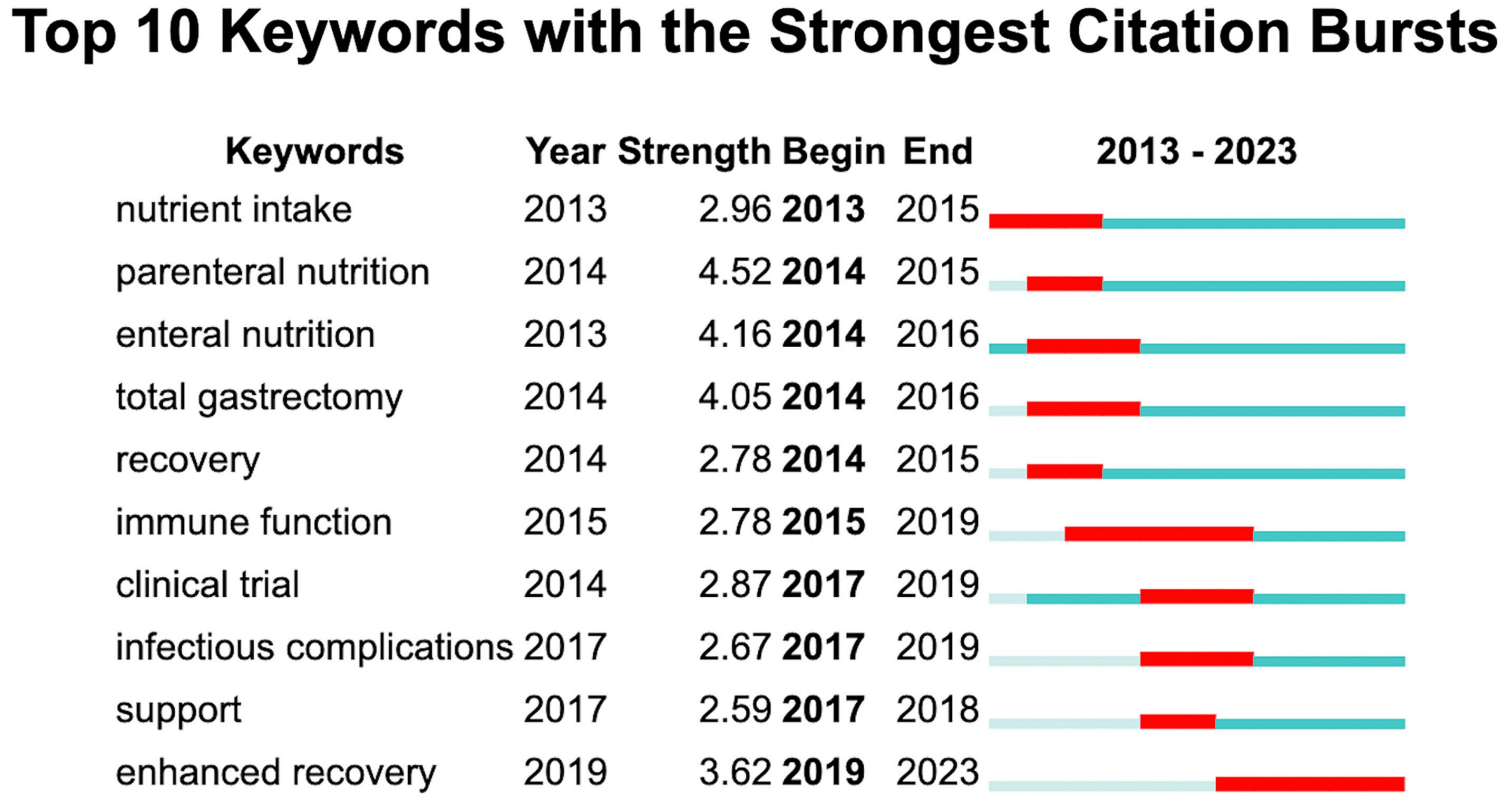
Top 10 keywords with the strongest citation bursts.

The keywords from 441 articles were analyzed to identify burst words. The 10 burst words with the highest strength are depicted in Figure 7. As illustrated, the focus on GC nutrition has evolved over the last decade from nutritional and surgical treatments to outcome-based treatments. Currently, nutrition-related enhanced recovery stands out as the current frontrunner.

### Documents

By setting the minimum citation count to 73, the top 10 most cited documents could be retrieved (Table 6), comprising 4 articles and 6 reviews. The predominant focus of these documents centered on investigating the impact of nutritional status and nutritional support on the prognosis of GC patients. The first high-cited document was published in Gastric Cancer and authored by Kamarajah et al. It is evident that the effect of nutrition on the prognosis of GC has been a focal point of research in this field over the past decade.

**Table 6:** The 10 most cited documents.

Rank	Title	Citations
1	Body composition assessment and sarcopenia in patients with gastric cancer: a systematic review and meta-analysis	164
2	The prognostic nutritional index is a predictive indicator of prognosis and postoperative complications in gastric cancer: a meta-analysis	155
3	Prevalence of malnutrition among gastric cancer patients undergoing gastrectomy and optimal preoperative nutritional support for preventing surgical site infections	152
4	Effectiveness of a preoperative exercise and nutritional support program for elderly sarcopenic patients with gastric cancer	150
5	The Impact of preoperative immune modulating nutrition on outcomes in patients undergoing surgery for gastrointestinal cancer: a systematic review and meta-analysis	126
6	Predictive potential of preoperative nutritional status in long-term outcome projections for patients with gastric cancer	113
7	Impact of malnutrition after gastrectomy for gastric cancer on long-term survival	92
8	Nutritional predictors for postoperative short-term and long-term outcomes of patients with gastric cancer	88
9	Dietary fiber intake reduces risk for gastric cancer: a meta-analysis	84
10	Clinical and immunological impact of early postoperative enteral immunonutrition after total gastrectomy in gastric cancer patients: a prospective randomized study	72

## Discussion

Over the past decade, researchers have conducted numerous studies in the field of nutrition in GC, focusing on nutrition support therapy and exploring the impact of nutritional status on prognosis prediction and recovery enhancement. Previous literature analyses have explored the ketogenic diet and fasting-mimicking diet (17, 18), but there is currently a lack of comprehensive articles specifically focused on nutrition in GC. This study analyzed 441 publications on GC nutrition from 2013 to 2023, revealing the evolution, current research status, hotspots, and potential trends of the field. It offers insights and avenues for future research in this important area.

The volume of publications can reflect the trend of a certain research field. Over the last decade, research in GC nutrition has undergone an evolution. From 2013 to 2019, the annual publications showed fluctuations, indicating that research encountered challenges during this period. However, the significant increase in publications from 2019 onward indicated substantial breakthroughs and advances such as enteral nutrition. Furthermore, the polynomial projection curve indicates an overall upward trend in annual publications, forecasting increased outputs in the coming years. This suggests that nutrition in GC will continue to garner more attention from researchers and experience further progress in the future.

The analysis of countries reveals leading nations in a research field. According to Table 1 and Figure 3, China, Japan, and South Korea emerge as the top three productive countries, underscoring their prominent role. This phenomenon may be attributed to the higher incidence of GC and the more complex dietary patterns in East Asia. Despite England having the highest number of citations, Figure 3 shows that East Asian countries, primarily led by China, have emerged as more influential in this field. Meanwhile, these countries have very close cooperative relationships with other nations. This indicates that cooperative and amicable relations between countries contribute to the publication of impactful research articles.

Analyzing institutional literature is key to identifying important institutions in a certain field. This study found that the Chinese Academy of Medical Sciences was the most productive institution, with Chinese organizations demonstrating higher productivity than other countries. Based on the overlay visualization of the institutions (Figure 4), it was clear that in the last decade, the core institutions had gradually shifted from the USA to China. The above-mentioned figure also showed that the institutions with the highest number of publications tended to have strong links with other institutions. In addition to this, literature from multi-center, multi-country collaborations is often considered more comprehensive and credible. These findings align with the analysis of countries mentioned above, demonstrating the significant potential of Asian countries in this research field.

As a basis for bibliometric analysis, Bradford’s law is beneficial in identifying the core, related, and peripheral journals within a certain field. The results showed that the most productive and influential journals in nutrition related to GC are all established journals of nutrition hold a significant position in both GC and nutrition. Reading articles from these journals can provide a better understanding of the findings in GC nutrition, as they are considered more authoritative. Thus, for those seeking comprehensive insights into nutrition related to GC, reading articles from the aforementioned journals is the most reliable approach.

Core authors are the primary driving force in a field. Daisuke Kobayashi and Yasuhiro Kodera, from Nagoya University Graduate School of Medicine, not only had the highest number of publications but also had the highest number of citations per article, making them undoubtedly the most productive and influential authors in the field. Their main research focus is on the impact of nutritional status on the prognosis of GC and other digestive tract tumors. Their study found that nutritional status could serve as a predictor of both long-term and short-term prognosis following GC surgery. Prognostic nutrition index (PIN) served as a significant predictor of postoperative morbidity, prognosis, and recurrence patterns of patients with stage II/III GC (19). Moreover, the preoperative controlling nutritional status (CONUT) score was an independent prognostic factor of overall survival among patients with stage 2 or 3 GC. Patients with a controlling nutritional status CONUT score of 2 or higher (CONUT-high group) were significantly older and had a worse Eastern Cooperative Oncology Group performance status, lower body mass index, and more advanced tumor-node-metastasis stage (20). In addition to this, they conducted a study on the effect of enteral nutrition on weight changes in patients after gastric surgery as well as a clinical trial of digestive fistula with different nutrients (21). All of the aforementioned directions represent prominent areas of nutritional research in postoperative GC patients.

Keywords reflect the key research directions in a certain field, and mapping their relationships can effectively categorize them into distinct directions (Figure 5). Nutritional oncology in GC encompasses four key research areas: perioperative and postoperative nutritional support (including enteral and parenteral nutrition), nutritional status assessment, complications, and prognosis. These topics represent classic research points in this field. Over the past decade, researchers have conducted extensive studies (11, 22). These studies have demonstrated the significant role that nutritional status plays in predicting the outcomes of patients with GC. Furthermore, the overlay visualization (Figure 6) also provided a gradual shift in research focus from surgical or post-treatment supportive care toward evaluating the influence of pre-treatment or on-treatment nutritional status on GC. In recent years, sarcopenia, skeletal muscle status, body composition, and malnutrition have emerged as prominent areas of interest in assessing the nutritional status of GC patients.

Burst words can serve as markers of current research focal points and are predictive of future trends in research directions. Nutrition research in the GC can be chronologically categorized into early and later research hotspots. In the early phase the predominant hotspots revolved around nutrition therapy particularly the impact of enteral and parenteral nutrients on GC patients undergoing surgery. In the later phase the hotspots were dominated by immune function management of complications and enhanced recovery. Since 2019 nutrition-related enhanced recovery has become a new hotspot. Research indicates that early implementation of nutritional support following GC surgery contributes significantly to enhanced recovery (23, 24). In general the research focus on nutritional status and its implications for prognosis in GC appears to be a promising trend.

Analyzing highly cited articles offers a comprehensive insight into the current perspectives and advancements within a particular field of study. By reading through the 10 most cited articles, we have gleaned important insights into nutritional considerations in GC. Malnutrition has been identified as a contributing factor to postoperative surgical site infections (25, 26). Conversely, preoperative nutrition support, such as immune-modulating nutrition, has shown potential to enhance patient prognosis (9). Sarcopenia, characterized by a progressive and generalized skeletal muscle disorder, is associated with an increased likelihood of adverse outcomes in cancer (27, 28). Body composition examination plays an important role in the assessment of a patient’s nutritional status, especially in the diagnosis of sarcopenia (28, 29). Studies also proved that GC patients diagnosed with sarcopenia preoperatively were more likely to encounter complications, while interventions such as preoperative exercise targeting sarcopenia have shown promise in improving prognosis (30, 31). With the release of the Global Leadership Initiative on Malnutrition (GLIM) standard, there is a substantial body of work in the field of nutrition for GC that remains to be undertaken (31, 32).

This study had several weaknesses. First, the search was conducted only in English, potentially excluding relevant literature published in other languages. Second, due to the database restrictions, the type of literature included reviews and articles, omitting case reports and case series. In addition, this study focused on discovering the evolution and frontiers of nutrition in GC in the last 10 years. The relatively short timeframe suggests that further exploration and extension of the study period would be beneficial. Although these limitations could potentially result in data loss, previous research has demonstrated that these shortcomings had minimal impact on the study outcomes (11, 33, 34).

## Conclusion

Nutrition in GC is a burgeoning area of research that is gaining increasing attention. Further investigation is necessary to enhance our understanding of how nutritional status impacts the prognosis of GC.

## Data Availability

The original contributions presented in the study are included in the article/supplementary material, further inquiries can be directed to the corresponding author.
